# Geographic disparities in the time to under-five mortality in Ghana

**DOI:** 10.1371/journal.pone.0291328

**Published:** 2023-09-12

**Authors:** Kamaldeen Mohammed, Abdul Razak Abubakari, Daniel Amoak, Roger Antabe, Isaac Luginaah

**Affiliations:** 1 Department of Geography and Environment, University of Western Ontario, London, Ontario, Canada; 2 Department of Population and Reproductive Health, University for Development Studies, Tamale, Ghana; 3 Department of Health and Society, University of Toronto Scarborough, Toronto, Ontario. Canada; Nelson Mandela University, SOUTH AFRICA

## Abstract

Globally, there has been tremendous advancement in medicine and child healthcare with increased life expectancy. That notwithstanding, the risk of under-five mortality ─ children dying before their fifth birthday remains relatively high in countries in Sub-Saharan Africa such as Ghana. In Ghana, under-five mortality remains a major public health problem that requires significant policy interventions. Using data from the 2017 Maternal Health Survey (n = 4785), we examined the geographic disparities in the time to under-five mortality in Ghana. The Kaplan Meier estimator showed significant (Log-rank: p< 0.001) rural-urban differences in the time to under-five mortality in Ghana. A disaggregated cox proportional hazards analysis showed that despite wide consensus that children in urban areas have a high survival rate, children in urban areas in northern regions of Ghana, especially the Upper West (HR = 4.40, p < 0.05) and Upper East (HR = 5.37, p < 0.01) Regions were significantly at increased risk of dying before the age of five compared to children in urban areas in the Greater Accra Region. A rural-urban comparison showed that children born in rural areas in all the other regions of Ghana were at a higher risk of dying before the age of five when compared to their counterparts in the rural areas of Greater Accra Region. Other factors such as sex of child, mothers’ age and use of the internet, number of household members, ethnicity and household wealth were significantly associated with the timing of under-five mortality in Ghana. Healthcare policies and programs such as immunizations and affordable child healthcare services should be prioritized especially in rural areas of regions with a high risk of child mortality. Also, there is a need to improve healthcare delivery in urban areas, particularly in northern Ghana, where deplorable healthcare service infrastructure and delivery coupled with high poverty rates put children at risk of dying before their fifth birthday.

## Introduction

Under-five mortality is an important public health indicator that measures the socio-economic status, health and quality of life of a country’s population [1]. According to the World Health Organization (WHO), under-five mortality constitutes the total number of deaths of children within the first five years of life per every 1000 live births [2]. Under-five mortality can be categorized into neonatal mortality (first 28 days after birth), infant mortality (first 12 months of life) and child mortality (first five years of life). Significant progress has been made towards the mitigation of under-five mortality. For instance, over the last three decades, the death of children before age five has declined by 60% globally, from 12.6 million in 1990 to 5.0 million in 2020 [3]. That notwithstanding, the magnitude of under-five mortality remains a great burden in sub-Saharan Africa and South Asia. These two regions account for more than 80 percent of the 5 million under-five mortalities recorded in 2020; sub-Saharan Africa and South Asia accounting for 2.8 million and 1.4 million deaths respectively [3, 4]. The WHO estimated that, in 2020, about half (47%) of under-five deaths occurred within the first 28 days of life (neonatal period), and Africa recorded the highest rate of neonatal mortality in the world with 43 percent of global newborn deaths (27 deaths per 1000 live births) [3]. Also, about a third of all neonatal deaths occur on the day of delivery and approximately three-fourth happens within the first week of life [5]. Most of these preventable deaths are linked to childbirth-related complications, preterm birth, congenital disabilities, diarrhea and infections, and are exacerbated by malnutrition and other socio-economic challenges [3, 5]. Consequently, eradicating under-five mortality is integral to the Sustainable Development Goals (SDGs), specifically SDG 3.2.

Previous studies have shown that socio-economic (i.e., age, education, wealth), cultural, biological, environmental and geographic factors mediate the experience and prevalence of under-five mortality [6–10]. For example, children of educated mothers have higher survival rates than children of mothers with no formal education [8]. Likewise, female children have a lower risk of infectious diseases and are more likely to survive beyond age five than male children [7, 9].

As countries work towards meeting the global target of the SDG 3.2 (i.e. to end preventable deaths of newborns and children under 5 years of age by 2030), 122 out of 195 countries have attained this target since 2019, and 20 nations have a high potential of achieving it by 2030, while 53 countries will need to intensify efforts to meet this target by 2030 [4]. Ghana is experiencing slow but steady progress towards this target through the provision of Community-based Health Planning and Service (CHPS) compounds, National Health Insurance Scheme (NHIS) and improved access to potable drinking water and sanitation [11–13]. However, under-five mortality is still a major concern in the country, with 45 deaths per 1000 live births as of 2020 [14]. Meanwhile, the global under-5 mortality rate in 2020 was 37 deaths per 1000 live births and that of Africa was 74 deaths [3]. A prior study involving 46 African countries affirmed that eight countries, including Ghana, are making very little effort to mitigate under five mortality [15].

In the Ghanaian context, a previous study explored the risk factors of under-five mortality in Accra using facility-based case control study design [16]. Also, [17] studied the key predisposing factors of under-five mortality in the Tano South District of Ghana; whiles [18] examined the association between under-five mortality and the usage of insecticide-treated bed net (ITN) among children in northern part of Ghana. Also, several studies examined the relationship between under-five mortality and factors such as individual and community determinants, socio-economic and demographic determinants, social and environmental risk factors [9, 19–21]. Other researchers have also employed data from the 2014 Ghana Demographic and Health Survey (GDHS) to examine the determinants of under-five mortality [9, 13, 17, 21]. Despite these important studies, what has not been examined is how geography, as a determinant of health, affects the timing to under-five mortality in Ghana. Yet this spatial knowledge would be relevant as Ghana strives to deploy crucial context-specific interventions/initiatives to meet SDG 3.2. Also, understanding how socio-economic factors affect the temporal and spatial nature of under-five mortality is essential in designing time-sensitive health policy programs. Hence, this study examined the geographic disparities in the time of under-five mortality in Ghana.

### The study context ─ Ghana

Ghana is a coastal and multi-cultural west African country geographically situated at latitude 7.9465° N, and longitude 1.0232° W with roughly 238, 537 km^2^ land area. The country is bordered to the North by Burkina Faso, to the East by Togo, the Atlantic Ocean to the South and Cote d’Ivoire to the West. The country’s landscape is a mix of lowlands and highlands, with a mean elevation of approximately 190 m. Though the country is currently made up of 16 administrative regions since 2018, they were 10 regions during the data collection for the Ghana Maternal Health Survey (GMHS) in 2017, namely; Western, Ashanti, Greater-Accra, Volta, Central, Brong-Ahafo, Northern, Upper East, Upper West, and Eastern. These 10 regions are used in our study.

**Fig 1:**
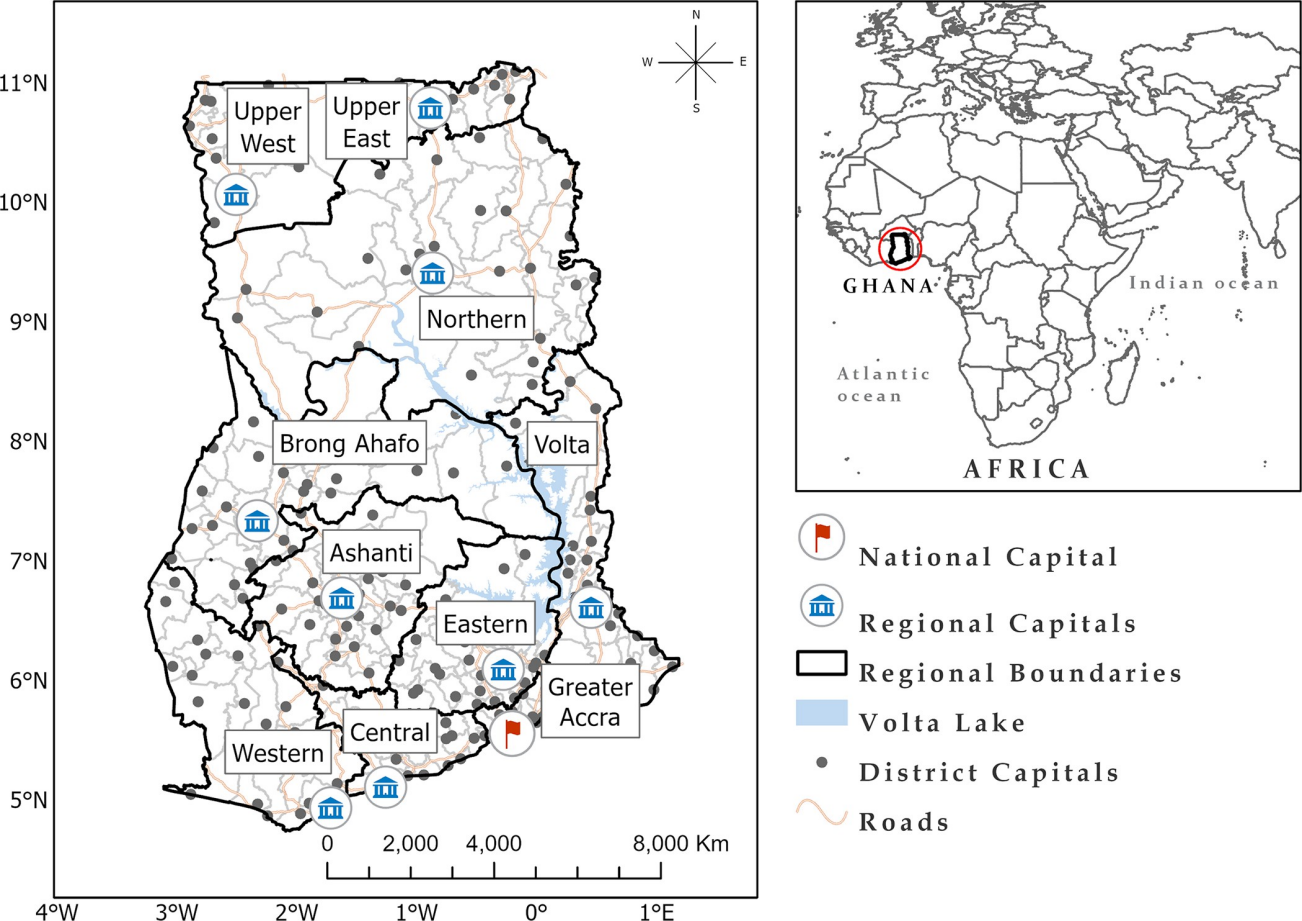
Map of Ghana showing the regional, district boundaries and regional capitals.

According to the Ghana Statistical Service (GSS), Ghana’s population is estimated at 30.8 million people, with females being the majority (50.7%) [22]. Averagely, each woman in Ghana has 4 children, varying from 3.1 in urban areas to 4.9 in rural areas [23]. Despite introducing several health policies and programs such as the NHIS and CHPS, maternal and child mortality remain a significant public health menace [23]. Ghana has a life expectancy of 63 (62.5 for males and 64.4 for women) and is ranked 155 in World Life Expectancy [24]. Also, there are both regional and residential disparities in socio-economic status [22] and diseases [25] in the country, which can affect child mortality rates. Regionally, poor education and poverty are widespread in northern Ghana compared to southern Ghana. Also, out of the 2857 healthcare facilities in Ghana, only 19% of these facilities are located and serve 5 regions in northern Ghana, however, the Greater Accra Region alone has a total of 438 health facilities [26]. Fig 1 shows a map of Ghana.

## Materials and methods

### Data

The study uses data from the 2017 Ghana Maternal Health Survey (GMHS), a nationally representative data on population and health. The study was implemented by the Ghana Statistical Service (GSS) in collaboration with the Ghana Health Service (GHS) from June 15, 2017 to October 12, 2017 [27]. The 2017 GMHS is the second survey since 2007 designed to collect data on child mortality, maternal health care, household characteristics, pregnancy history and verbal autopsy in national, regional, and rural and urban areas in Ghana. The sample design was based on the 2010 Ghana Population and Housing Census sampling strategy [27]. First, 466 urban and 434 rural cluster samples were selected [27]. Second, 30 households were selected in each cluster, making a total sample of 27,000. Women aged 15–49 were selected for interviews in each household.

### Measures

The GMHS collected data on 4785 children who died (i.e., death of any cause) before their 5th birthday during interviews with mothers. The primary outcome variable in the study is under-five mortality—the probability of a child dying between 0 to 59 months upon birth. Women aged 15–49 were asked to indicate the age at which their child/ren died (i.e., if they had a child who died). The time (age of death in months) variable was computed from this question.

The main explanatory variable was the region of residence. These included (0 = Greater Accra, 1 = Western, 2 = Central, 3 = Volta, 4 = Eastern, 5 = Ashanti, 6 = Brong Ahafo, 7 = Northern, 8 = Upper East and 9 = Upper West). The Greater Accra Region is the national capital and was therefore made the reference region for comparison of the geographic disparities in under-five mortality. We also selected other contextually relevant covariates based on previous studies [9, 28, 29]. These included residence (0 = rural, 1 = urban); Mothers age; sex of child (0 = male, 1 = female); mothers education (0 = formal education, 1 = no formal education); ethnicity (0 = Akan, 1 = Ga/Dangme, 2 = Ewe, 3 = Guan, 4 = Mole-Dagbani, 5 = Grusi, 6 = Gurma, 7 = Mande and 8 = Other ethnicities); child health insurance coverage (0 = yes, 1 = no); mother’s birth parity (i.e., total number of births including live births and stillbirths); mother’s access to internet (0 = access to internet, 1 = no access); number of co-wives in household; number of household members and household wealth index (0 = richest, 1 = richer, 2 = middle, 3 = poorer, 4 = poorest). The household wealth index is a standardized measure of wealth used by the DHS, usually in the Global South, where income data is unavailable or unreliable. The wealth index is computed using a composite cumulative living standard of each household based on ownership of selected assets. These assets include mobile phones, televisions, radio, tractors, motorbikes, bicycles, vehicles, livestock, access to water, household building materials etc. Principal Component Analysis is used to transform these household assets into a continuous scale of relative wealth, which is further categorized into five distinct wealth categories (i.e., richest, richer, middle, poorer and poorest). For more detailed steps on creating the wealth index using the DHS guidelines, see [30].

### Analysis

Data analysis was done in R version 4.1.2. We used the ’*Survey’*, ’*Survival’* and *’Survminer’* libraries in R in implementing the analytical approaches. First, univariate descriptive statistics were used to understand the distribution of the sample population. Categorical data were summarized using frequencies and percentages, while means, minimum and maximum values were reported for continuous variables. The Kaplan Meier Estimator and cox proportional hazard model were used to understand the geography of timing to under-five mortality. The experience and risk of under-five mortality from the Kaplan Meier estimator were linked to spatial data of the various regions in Ghana, allowing the visualization of spatio-temporal nature of under-five mortality in the regions at a time interval of 12 months (a year). The cox proportional hazards Ratio was used to understand the predictors of timing to under-five mortality in Ghana. The bivariate and multiple cox proportional hazards Ratio analysis was disaggregated into three models, rural, urban, and a combination of rural and urban samples to understand the nuances in the geography of timing to under-five mortality in Ghana. To account for the complex survey design of the DHS data, we used the complex survey functions in R. For descriptive statistics, we used the command ‘*wpct*’ to generate weighted tables of the percentage of data in each category of all variables. The function ‘*svyk*’ was used to estimate the survival function for the weighted Kaplan-Meir estimator. Finally, we used the function ‘*svycoxph*’ to estimate the bivariate and multiple survey-weighted cox models.

### The Kaplan Meier estimator

The Kaplan Meier estimator, a nonparametric measure, was used to estimate the survival probabilities of children using their age at death (before age 5) as the survival time. The equation for the Kaplan Meier estimator is given here in Eq 1. Given that *k* participants (i.e., children) have experienced events (i.e., death) in times (i.e., age at death) *t1<t2<t3<t4<t5<⋯<tk*. The probability of surviving from one time to the other can be multiplied to compute the cumulative survival probability. The survival probability at time *tj*, *S(tj)*, is computed from *S(tj-1)* the probability of being alive at *tj-1*, *nj* the number of participants alive before *tj* and *dj* the number of events at *tj*. The log-rank test was also used to test for significant regional and rural-urban differences in timing to under-five mortality.


Ŝ(tj)=S(tj−1)(1−djnj) (1)

#### The cox proportional hazards model

The cox proportional hazard model is a semi-parametric function that models the hazard as a function of other explanatory variables. The equation for the cox proportional hazards model is given as

$$h(t)=h_{0}(t)exp\{\beta_{1}x_{1}+\beta_{2}x_{2}+\cdots +\beta_{p}x_{p}\}$$ (2)

Where *β*_1_, *β*_2_,…..*β*_*p*_ are parameter estimates that measure the effect of the risk factors X_1_, X_2_, …, X_p_ on the logarithm of the ratio of the death hazard to the baseline hazard function. The baseline hazard here refers to a participant with zero values for all X-variables. We reported unadjusted and adjusted hazard ratios derived from the cox proportional hazards regression. The Schoenfeld global and individual covariate test was used to diagnose the model fit for the proportional odds assumption of the cox regression.

## Results and discussions

### Descriptive statistics

Table 1 provides the descriptive results from the analysis. About 34% of the surveyed participants resided in urban centres and 66% resided in rural areas. For regional distribution, 4% lived in the Greater Accra Region, 8% in Western Region, 7% in Central Region, 5% in Volta, 6% in Eastern, 9% in Ashanti, 8% in Brong Ahafo, 25% in Northern Region, 11% in Upper East Region and 16% in the Upper West Region. The results further showed the ethnic diversity of Ghana where 27% were Akans, 40% were Mole-Dagbani, 11% were Gurma, 7% were Ewe, 5% were Grusi, 4% were Guan, 3% were Ga/Dangme and 2% were other ethnic groups. The minimum number of household members was 1 with some households having up to 32 members.

**Table 1:** Descriptive statistics of study sample.

Variables		Frequency	Mean (min-max)	Percentage%
**Residential type**				
Urban		1638		34
Rural		3147		66
**Region of residence**				
Greater Accra		208		4
Western		360		8
Central		330		7
Volta		259		5
Eastern		307		6
Ashanti		443		9
Brong Ahafo		394		8
Northern		1182		25
Upper East		526		11
Upper West		776		16
**Mother’s Age**			38.56(15–49)	
**Sex of Child**				
Male		2614		55
Female		2171		45
**Mother’s education status**				
Formal education		2051		43
No formal education		2734		57
**Ethnicity**				
Akan		1301		27
Ga/Dangme		120		3
Ewe		346		7
Guan		181		4
Mole-Dagbani		1921		40
Grusi		234		5
Gurma		532		11
Mande		55		1
Other		95		2
**Child insurance coverage**				
Yes		3411		88
No		469		12
**Mother’s birth parity**			5.78(1–14)	
**Mother’s access to internet**				
Yes		225		5
No		4560		95
**Number of co-wives in household**			2.34(2–5)	2.34
**Number of household members**			6.38(1–32)	
**Household wealth**				
Richest		311		6
Richer		574		12
Middle		747		16
Poorer		1079		23
Poorest		2074		43
	Total sample: 4785			

Furthermore, the results indicated that 6% of households were within the richest wealth quintile, 12% in the richer, 16% in middle, 23% in poorer and 43% in the poorest. In addition, 55% of children were male and 45% were female. Among all children, 88% had health insurance coverage and 12% had no health insurance coverage, only 5% of mothers had access to the internet and 95% did not have internet services. The minimum birth parity was 1 with a maximum of 14 children. The age range of participants was between 15 to 49 years old. Finally, only 43% of mothers had formal education.

### Residential differences in timing to under-five mortality

**Fig 2:**
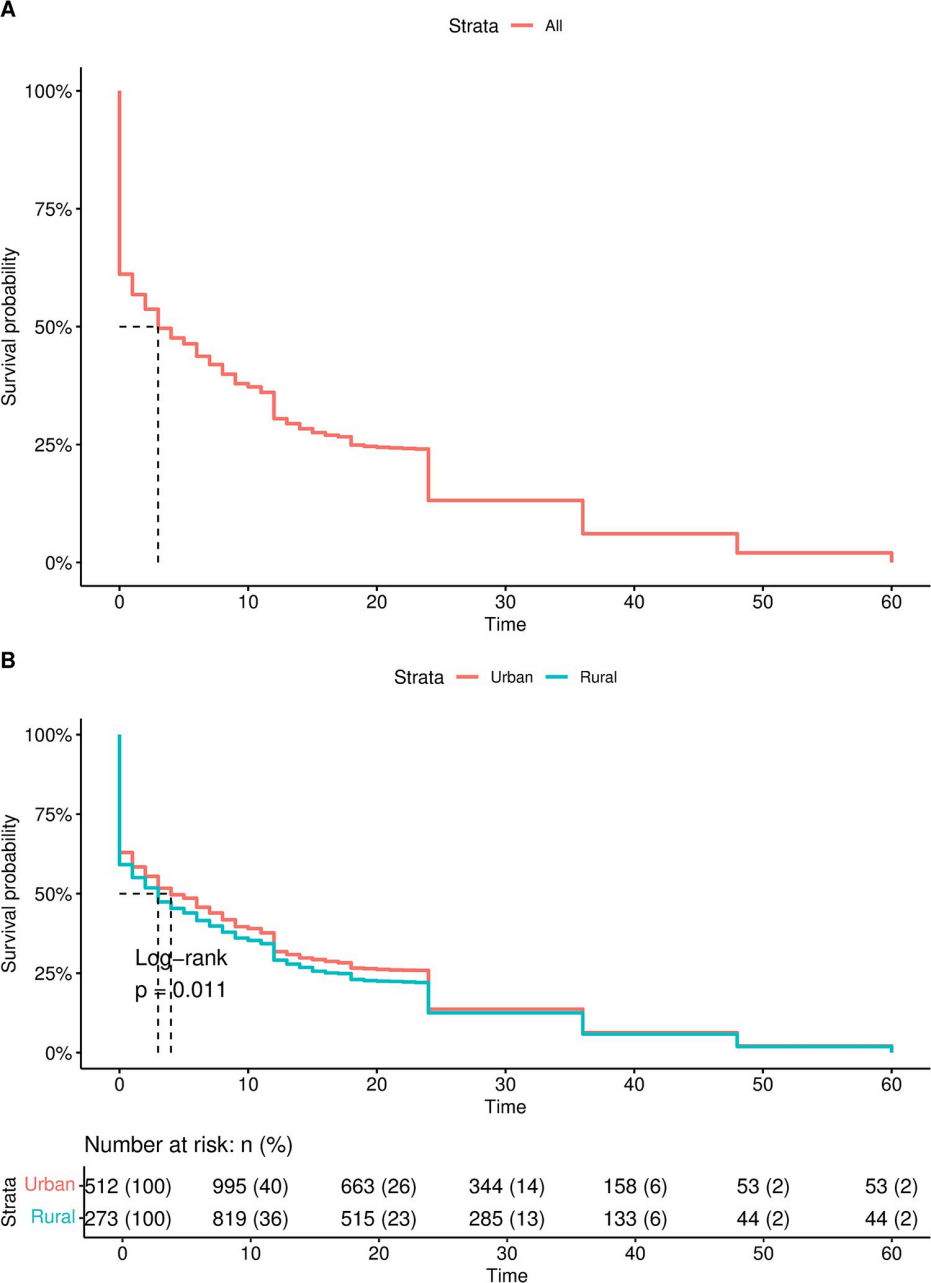
General and rural-urban Kaplan Meier survival curves of under 5 mortalities.

The Kaplan Meier survival curves in Fig 2 visualize the life table of children in Ghana. The horizontal axis shows the time to under-five mortality in months at death, and the vertical axis shows the survival probabilities. Fig 2A indicates that at time 0, the survival rate is 100% (i.e., all children are alive). As the age of the children increases, the likelihood of the children surviving beyond 60 months (5 years) decreases. Though this is the general outlook, Fig 2B shows significant differences (Log-rank: p< 0.05) in under-five mortality between rural and urban dwellers. The survival rates between rural and urban dwellers differ between 0 to 25 months but converge above 25 months (i.e., after 2 years). S1 Table in the appendix shows detailed risk and survival rates of children per year or 12 months.

### Regional differences in timing to under-five mortality

**Fig 3:**
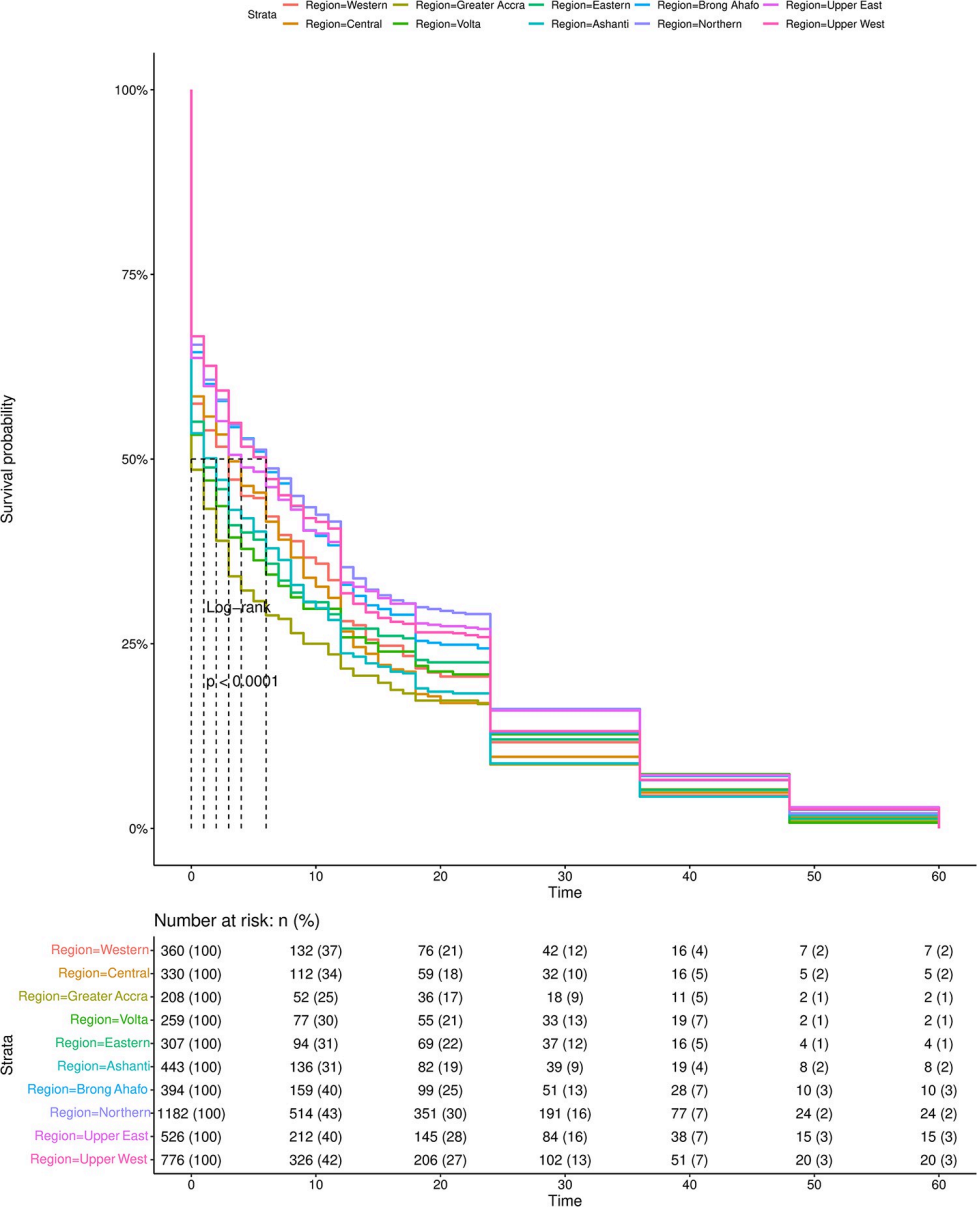
Kaplan Meier curve of under 5 mortalities by different regions in Ghana.

Fig 3 is a Kaplan Meier curve that shows significant differences (Log-rank: p< 0.001) in the survival rates of children in the different regions of Ghana. At time 0, all regions have a survival rate of 100%. However, significant differences in regional under 5 survival rates begin to emerge until approximately time 37 (i.e., children are 37 months old) when regional under-five mortality converge and show more negligible differences in regional survival rates. The forest plot in Fig 3 further shows the bivariate cox proportional hazard ratio. S2 Table in the appendix shows more detailed regional risk and survival rates of under 5 mortality every 12 months.

**Fig 4:**
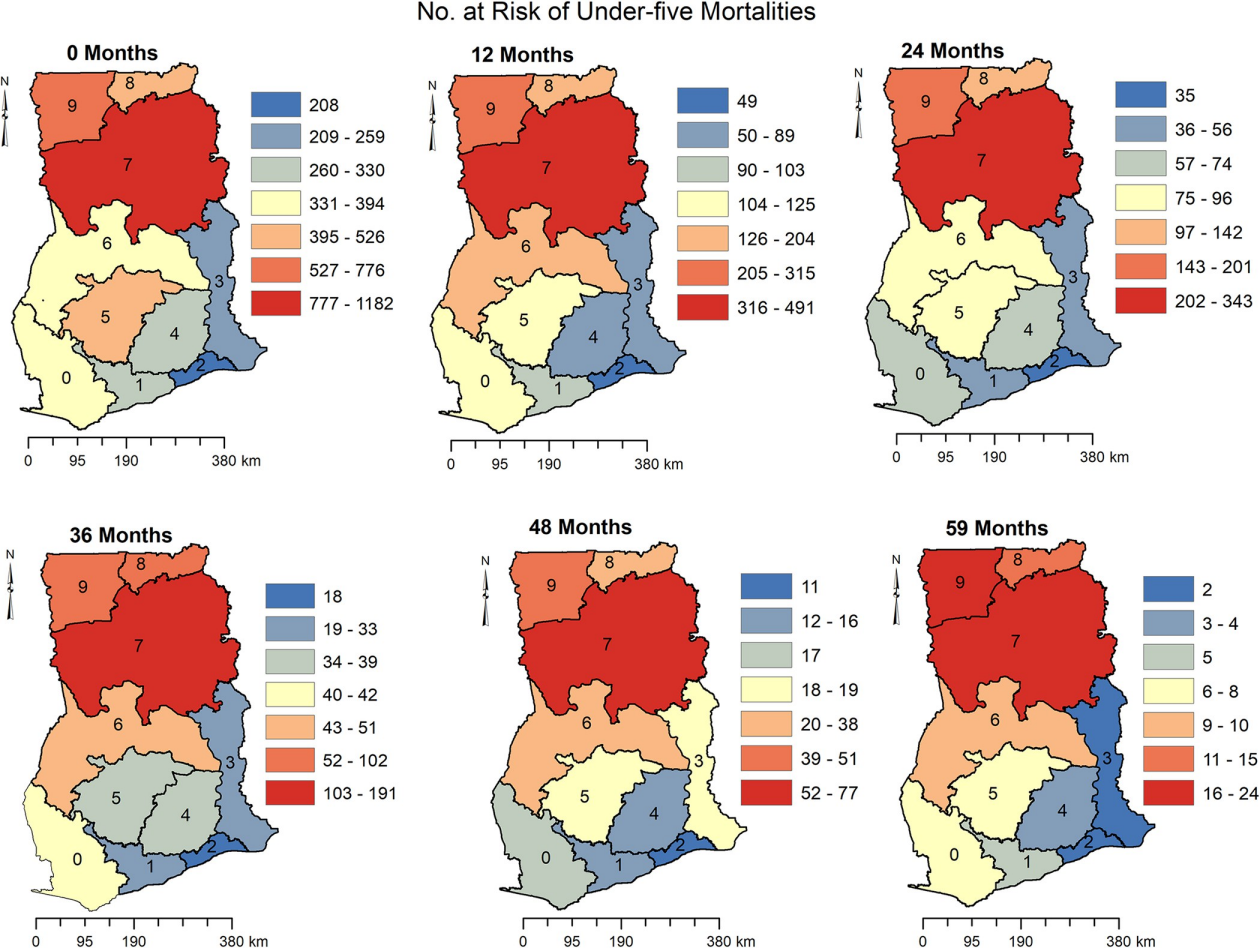
Spatio-temporal distribution of the number of children at risk of dying before the age of 5 in Ghana. (0) Western Region, (1) Central Region, (2) Greater Accra Region, (3) Volta Region, (4) Eastern Region, (5) Ashanti Region, (6) Brong Ahafo Region, (7) Northern Region, (8) Upper East Region, (9) Upper West Region.

Fig 4 shows the regional distribution of risk to under 5 mortalities at 0, 12, 24, 36, 48 and 59 months in Ghana. Children in Northern Region have consistently had the highest risk of dying before age 5. Similarly, children in the Upper West and Upper East Regions had a higher risk of dying before age 5. Children in the Greater Accra Region also consistently had the lowest risk of under 5 mortalities at 0, 12, 24, 36, 48, and 59 months. Aside from the Greater Accra Region, children in Western and Eastern Regions also had a relatively lower risk of dying before age 5.

**Fig 5:**
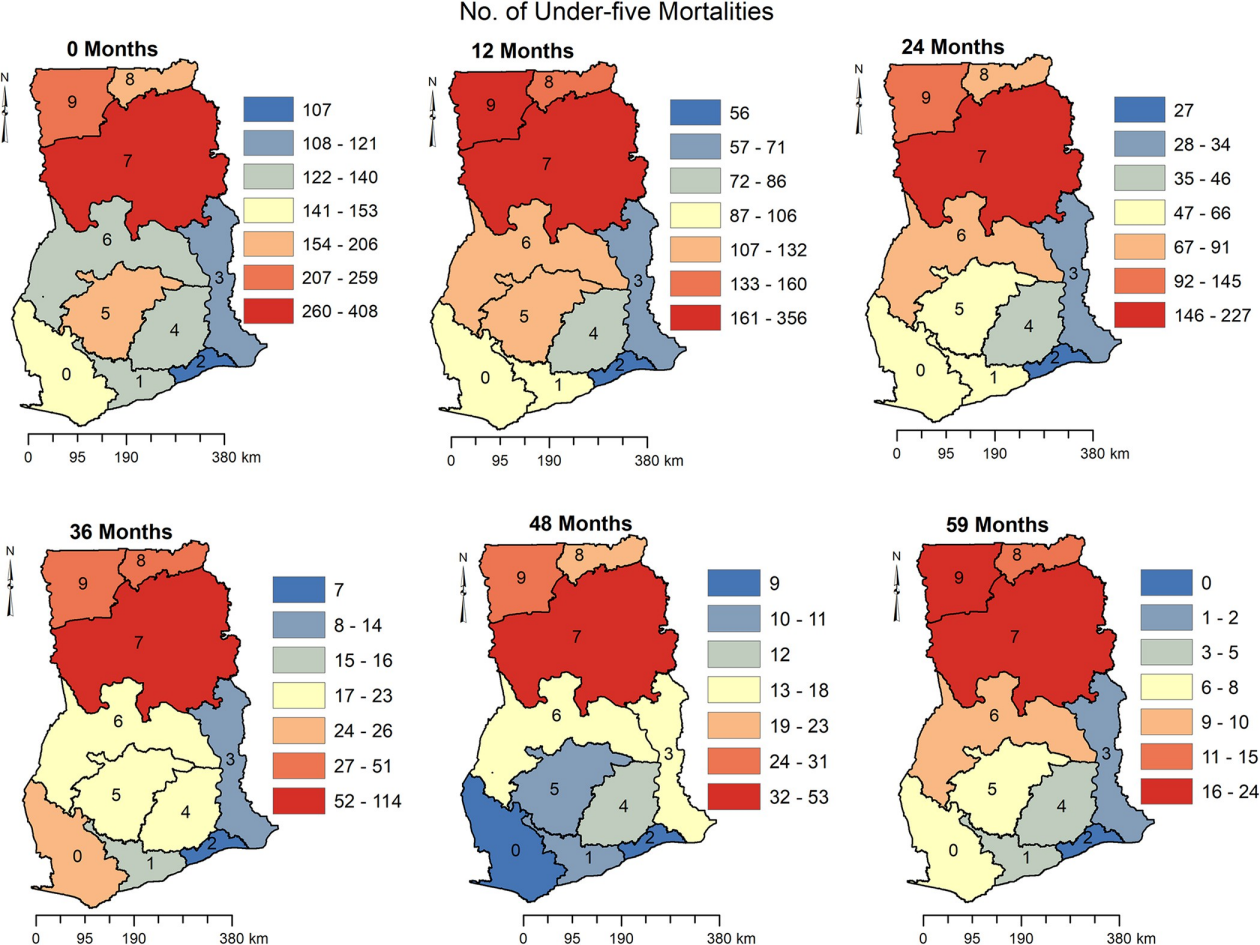
Spatio-temporal distribution of the number of children who died before the age of 5 in Ghana. (0) Western Region, (1) Central Region, (2) Greater Accra Region, (3) Volta Region, (4) Eastern Region, (5) Ashanti Region, (6) Brong Ahafo Region, (7) Northern Region, (8) Upper East Region, (9) Upper West Region.

Fig 5 shows the number of children that died before age 5 at 0, 12, 24, 26, 48 and 59 months. The observed number of children who died before the age of 5 followed a similar pattern to the number of children at risk of dying before the age of 5 as seen in Fig 5. For example, 260–400 children died when they were less than a month old in the Northern Region of Ghana, 161–350 children died at the age of 1 year (12 months), 147–227 died at the age of 2 years (24 months), 52–114 at age 3 (36 months), 32–53 at age 4 (48 months) and 16–24 a month before the age of 5. Observed under 5 mortality was lower in the Greater Accra Region compared to other regions.

### Bivariate cox regression model of time to under-five mortality

The bivariate cox proportional hazard ratio in Table 2 shows significant differences in the risk of under-five mortality. The risk of under-five mortality was lower in the Upper West region (HR = 0.73, p-value < 0.001), Upper East region (HR = 0.73, p-value < 0.001), Northern region (HR = 0.71, p-value < 0.001), Brong Ahafo region (HR = 0.74, p-value < 0.001), and Western region (HR = 0.84, p-value < 0.05) compared to Greater Accra region. A bivariate cox proportional hazard ratio was ran separately for rural under-five mortality, and the results were largely consistent with the general risk of under-five mortality with a few changes. Children in urban areas in the Upper West region (HR = 0.90, p-value < 0.01), Upper East region (HR = 0.74, p-value < 0.05), Northern region (HR = 0.69, p-value < 0.001), Brong Ahafo region (HR = 0.71, p-value < 0.01), and Volta region (HR = 0.74, p-value < 0.05) had lower risk of dying before the age of 5 when compared to children in urban areas of the Greater Accra region, as seen in model 2. However, no significant regional differences were observed in the risk of under-five mortality in rural areas at the bivariate level.

**Table 2:** Bivariate cox proportional hazards regression.

Variables	Model 1 (General)	Model 2 (Urban)	Model 3 (Rural)
	**HR(SE)**	**HR(SE)**	**HR(SE)**
**Region of residence**			
Greater Accra	1.00	1.00	1.00
Western	0.84(0.10)*	0.83(0.09)	0.74(0.14)
Central	0.87(0.10)	0.88(0.09)	0.76(0.14)
Volta	0.87(0.10)	0.74(0.11)*	0.87(0.14)
Eastern	0.87(0.11)	0.83(0.10)	0.73(0.14)
Ashanti	0.91(0.10)	0.93(0.08)	0.78(0.14)
Brong Ahafo	0.74(0.10)***	0.71(0.10)**	0.67(0.14)
Northern	0.71(0.09)***	0.69(0.11)***	0.63(0.14).
Upper East	0.72(0.09)***	0.74(0.20)*	0.63(0.15).
Upper West	0.73(0.09)***	0.90(0.20)	0.64(0.15)
**Mother’s Age**	0.99(0.00)***	0.99(0.00)**	0.99(0.00)***
**Sex of Child**			
Male	1.00	1.00	1.00
Female	0.93(0.03)**	0.90(0.05).	0.91(0.04)*
**Mother’s education status**			
Formal education	1.00	1.00	1.00
No formal education	0.81(0.03)***	0.76(0.06)***	0.86(0.04)***
**Ethnicity**			
Akan	1.00	1.00	1.00
Ga/Dangme	1.01(0.12)	0.84(0.10)	1.26(0.11)
Ewe	1.04(0.07)	0.88(0.08)	1.23(0.06).
Guan	0.88(0.08)	0.93(0.13)	0.95(0.10)
Mole-Dagbani	0.79(0.04)***	0.73(0.07)***	0.86(0.05)**
Grusi	0.86(0.08).	0.81(0.18)	0.99(0.10)
Gurma	0.81(0.05)***	0.66(0.11)**	0.91(0.06)
Mande	0.78(0.12)*	0.74(0.26)	0.93(0.21)
Other	1.07(0.11)	1.22(0.14)	1.15(0.17)
**Child insurance coverage**			
Yes	1.00	1.00	1.00
No	0.98(0.06)	1.00(0.08)	0.98(0.06)
**Mother’s birth parity**	0.97(0.01)***	0.95(0.01)***	0.98(0.01)*
**Mother’s access to internet**			
Yes	1.00	1.00	1.00
No	0.64(0.09)***	0.59(0.07)***	0.94(0.12)
**Number of co-wives in household**	0.99(0.01)	1.00(0.04)	0.93(0.08)
**Number of household members**	0.99(0.00)**	0.96(0.01)***	0.99(0.01)
**Household wealth**			
Richest	1.00	1.00	1.00
Richer	0.72(0.08)***	0.73(0.07)***	0.7(0.14)
Middle	0.71(0.08)***	0.69(0.07)***	0.72(0.13)
Poorer	0.62(0.07)***	0.6(0.08)***	0.66(0.13)*
Poorest	0.61(0.07)***	0.67(0.14)**	0.62(0.13)*

*P<0.05

**P<0.01

***P<0.001, HR = Hazard Ratio, SE = Standard Error

At the bivariate level, other significant factors that predicted survival probabilities included mother’s age, sex of child, mother’s education, ethnicity, mother’s birth parity, access to internet, number of household members, and household wealth. Increased age of mothers (HR = 0.99, p-value < 0.001) was associated with lower risk of children dying before age 5. Girls had lower risk probabilities (HR = 0.93, p-value < 0.01) of dying before the age of 5 compared to boys. Also, children whose mothers had no formal education (HR = 0.81, p-value < 0.001) had lower risk of mortality before the age of 5 than children of mothers who had formal education. This result was consistent across rural (HR = 0.86, p-value < 0.001), and urban (HR = 0.76, p-value < 0.001) areas across all the regions in Ghana. Ethnicity was also significantly associated with the survival probabilities of children under-five. Children that belonged to the Mole-Dagbani (HR = 0.79, p-value < 0.001), Gurma (HR = 0.81, p-value < 0.001), and Mande (HR = 0.78, p-value < 0.05), ethnic groups had lower risk of mortality compared to children that belonged to the Akan ethnicity. These results were also consistent in urban centers across regions. Children in the Mole-Dagbani and Gurma ethnicity in urban areas had a lower mortality risk than children of Akan ethnicity in urban areas. Other significant factors that predicted the survival probabilities of under-five children included access to the internet, number of household members and household wealth, as indicated in Table 2.

### Multiple cox regression model of timing to under-five mortality

The multiple cox regression model showed regional differences in risk of timing to under-five mortality in Table 3. In Model 4, we found that children in other regions had an increased risk of under-five mortality compared to children in the Greater Accra Region. For example, children in the Upper East Region (HR = 2.29, p < 0.05), Northern Region (HR = 2.08, p < 0.05), Central Region (HR = 2.18, p < 0.05), Western Region (HR = 2.32, p < 0.05) had higher risk of under 5 mortalities compared to children in the Greater Accra Region. More so, a segregated urban-rural analysis further revealed that urban centers in the Upper West Region (HR = 4.40, p < 0.05), Northern Region (HR = 3.43, p < 0.1), Volta Region (HR = 3.08, p < 0.1) and Upper East Region (HR = 5.37, p < 0.01) were at higher risk of dying before the age of 5 compared to children in urban centers in the Greater Accra Region as seen in Model 5. The differences in under 5 mortalities across rural areas in the regions were even more obvious. Children in rural areas in all the regions except Eastern Region were significantly at a higher risk of dying before the age of 5 than children in rural areas in the Greater Accra Region, as seen in Model 6 of Table 3.

**Table 3:** Multiple cox proportional hazards regression.

Variables	Model 4 (General)	Model 5 (Urban)	Model 6 (Rural)
	HR(SE)	HR(SE)	HR(SE)
**Region of residence**			
Greater Accra	1.00	1.00	1.00
Western	2.32(0.40)*	3.06(0.71)	4.81(0.73)***
Central	2.18(0.39)*	2.25(0.63)	7.01(0.75)***
Volta	1.51(0.36)	3.08(0.51).	3.03(0.71)**
Eastern	1.23(0.39)	2.61(0.75)	2.2(0.68).
Ashanti	1.53(0.39)	1.52(0.57)	4.28(0.71)***
Brong Ahafo	1.42(0.38)	2.53(0.59)	3.35(0.72)**
Northern	2.08(0.37)*	3.43(0.59).	4.39(0.71)***
Upper East	2.29(0.38)*	5.37(0.70)**	4.79(0.73)***
Upper West	1.77(0.37)	4.40(0.71)*	3.5(0.72)**
**Mother’s Age**	0.99(0.01)**	0.97(0.02)*	0.98(0.01)*
**Sex of Child**			
Male	1.00	1.00	1.00
Female	0.88(0.06)*	0.71(0.19)*	0.93(0.09)
**Mother’s education status**			
Formal education	1.00	1.00	1.00
No formal education	1.04(0.09)	1.09(0.23)	1.24(0.13)
**Ethnicity**			
Akan	1.00	1.00	1.00
Ga/Dangme	3.16(0.34)***	1.69(0.60)	9.83(0.72)***
Ewe	1.35(0.20)	0.89(0.46)	1.53(0.24).
Guan	1.08(0.18)	1.34(0.64)	0.92(0.27)
Mole-Dagbani	0.95(0.15)	0.48(0.44)*	0.93(0.20)
Grusi	1.29(0.19)	1.38(0.52)	1.27(0.30)
Gurma	1.27(0.17)	0.74(0.46)	1.15(0.22)
Mande	0.96(0.28)	0.52(0.78).	0.92(0.44)
Other	1.56(0.21)*	0.72(0.63)	1.71(0.31).
**Child insurance coverage**			
Yes	1.00	1.00	1.00
No	0.94(0.11)	1.53(0.29)	0.84(0.15)
**Mother’s birth parity**	1.01(0.02)	1.05(0.06)	1.02(0.03)
**Mother’s access to internet**			
Yes	1.00	1.00	1.00
No	1.51(0.20)*	2.07(0.48)*	1.45(0.31)
**Number of co-wives in household**	1.00(0.00)	1(0.05)	1.01(0.10)
**Number of household members**	1.01(0.01)	1.06(0.03)*	0.99(0.01)
**Household wealth**			
Richest	1.00	1.00	1.00
Richer	0.79(0.22)	0.43(0.36)**	0.77(0.45)
Middle	0.84(0.23)	0.41(0.37)*	0.96(0.43)
Poorer	0.77(0.21)	0.28(0.40)***	0.83(0.42)
Poorest	0.77(0.21)	0.39(0.49)*	0.8(0.42)

^●^P< 0.1, *P<0.05

**P<0.01

***P<0.001, HR = Hazard Ratio, SE = Standard Error

Other factors were significantly associated with the risk of under-five mortality. An increase in the age of mothers (HR = 0.99, p < 0.01) was significantly associated with a lower risk of under-five mortality. This result was consistent in urban (HR = 0.97, p < 0.05) and rural (HR = 0.98, p < 0.05) areas. Also, ethnicity was significantly associated with the risk of under-five mortalities. Children from the Ga/Dangme (HR = 3.16, p < 0.001) and other ethnicities (HR = 1.56, p < 0.05) had an increased risk of dying before the age of 5 compared to children of Akan ethnicity. The ethnic disparity in under-five mortality was most visible within rural areas. Children in rural areas of Ga/Dangme ethnicity had an even higher (HR = 9.83, p < 0.001) risk of dying before age 5 compared to children of rural areas and of Akan ethnicity. Access to internet (HR = 1.51, p < 0.05) was also significantly associated with under 5 mortalities, especially in urban areas (HR = 2.07, p < 0.05). Counterintuitively, children in the richer (HR = 0.43, p < 0.01), middle (HR = 0.41, p < 0.05), poorer (HR = 0.28, p < 0.001) and poorest (HR = 0.39, p < 0.05) households were at lower risk of under 5 mortalities in urban centers compared to the richest as seen in Model 5. Also, larger household sizes (number of people in the house) were associated with a higher (HR = 1.06, p < 0.05) risk of under 5 mortalities in urban centers.

## Discussion

This paper explored the geographic disparities in time to under-five mortality in Ghana. Our findings revealed regional and residential differences in the survival rates of children under five in Ghana. Children under-five years old residing in rural and urban areas of all other regions but the Greater Accra, had a lower survival probability than their counterparts in the Greater Accra region. Factors such as sex of children, household wealth, internet use, and ethnicity were significant determinants of the rural-urban dynamics in the time to under-five mortalities in Ghana. Given the importance of geographical context in health policy planning and deployment, this paper contributes to the literature on the spatio-temporal and socio-economic determinants of child health in Ghana and similar context in SSA.

The findings revealed regional and residential disparities in under-five mortality in Ghana, consistent with earlier studies [6, 12, 29, 31] that show rural-urban differences in under-five mortality. Children living in the Upper East, Northern, Central and Western Regions had a higher risk of dying before age 5 than those living in the Greater Accra Region. These findings may not be too surprising given the colonial and post-colonial legacies of Ghana’s healthcare system, where healthcare facilities and services are concentrated within the national capital, Greater Accra region, and other urban areas [32]. For instance, [9, 33] allude to persistent regional disparities in the distribution of healthcare services, policy and economic resources in favour of the Greater Accra region and other urban centres in Ghana. Consequently, children living in regions with inadequate and under-resourced healthcare services may succumb to preventable health complications at a young age.

A further rural-urban disaggregated analysis showed that children living in urban areas in the Northern Regions (i.e., Upper East, Upper West and Northern) had higher risk of dying before age 5 compared to children living in urban areas in Greater Accra, the national capital. This shows that although children in urban areas generally have a higher survival probability than children in rural areas [6, 12, 29, 31], this may not be true for all urban areas. Particularly for children living in urban areas in Northern Ghana (Upper East, Upper West, Northern and Volta Regions), contextual and other intersecting socio-economic, cultural and environmental factors may make them still vulnerable to under-five mortality compared to children in other regions. Northern Ghana is comparatively undeveloped, with poor healthcare infrastructure and services, even in urban areas [31, 33]. For instance, only 19% of all healthcare facilities in Ghana serve the five regions in the northern part, with the Tamale Teaching Hospital being the only referral center, serving about 6 million people in the regions [22, 26]. That notwithstanding, the hospital is woefully under-resourced with inadequate healthcare services and personnel compared to other referral hospitals in southern Ghana. Also, the three northern regions have consistently remained the most economically impoverished regions in Ghana [22]. The poor state of healthcare service infrastructure, coupled with mass impoverishment, means that children in Northern Ghana, including those in urban areas, would likely die from curable diseases and health complications that can be addressed in areas in Southern Ghana.

Also, the regional disparities in under-five mortality were more noticeable in rural areas across Ghana. Children living in rural areas in all the regions but Eastern had a higher risk of dying before the age of five compared to children living in rural areas in the Greater Accra Region. Despite the general vulnerabilities of rural areas to under-five mortality [29], children in rural areas in the Greater Accra region may have proximate access to better healthcare services, thus, better survival chances than children in rural areas in the other regions. For example, rural areas in Greater Accra Region may receive timely medical supplies and health care personnel and specialist earlier than rural areas in other regions farther from the national capital. Given these dynamics, children in rural areas in Northern Ghana are particularly at high risk as they are the farthest away and do not enjoy the geographic privileges to healthcare access compared to areas close to the national capital.

The findings showed that factors such as sex of a child, mother’s use of internet, ethnicity, household size and wealth were significant determinants of time to under-five mortality in Ghana. Female children had a slower time to under-five mortality than boys, which is consistent with the literature [34, 35]. The sex differences in under-five mortality can be explained by socio-economic, biological, and environmental factors [36]. For example, [31] argue that male children may experience an increased risk of under-five mortality because they have a lower infection resistance, elevated risk of premature birth, and averagely, larger body size and head, which may create complications during childbirth. Furthermore, others have argued that male children are genetically susceptible to congenital malformations of the urogenital system and acute lung respiratory infections, which are associated with high mortality rates [37]. Socio-cultural factors such as gender discrimination and sex preferences also partly explain the unequal vulnerability to under-five mortality between male and female children [38]. The findings further suggested that children of mothers who did not use the internet had a faster time to under-five mortality than children of mothers who used the internet, especially in urban areas. This finding concurs with [39, 40], reiterating the facilitating role of the internet in access to child health and nutrition information. However, this was not statistically significant in rural areas possibly due to the limited internet infrastructure. The internet can be a vital source of information on child hygiene and diets, thus, increasing the chances of child survival. In this context, urban mothers who use the internet may improve their knowledge on child hygiene and healthy foods for children [41, 42]. For instance, [40] noted that women who patronized an internet-based infant feeding practices were more likely to practice exclusive breastfeeding, which can reduce infant mortality. Consistent with the existing literature, our findings further demonstrated that as mothers aged, their children had a lower risk of dying before their fifth birthday [43]. Compared to younger mothers, older mothers often tend to be experienced with childcare that works to increase their children’s survivability past age five.

Children in lager households had a faster time to under-five mortality, particularly in urban areas. Amid limited financial resources, high cost of living in urban areas and inadequate healthcare services [33, 44], increasing household members may translate to a high dependency ratio and competition over limited resources including the health and dietary needs of children. Ethnic affiliation was also associated with the timing of under-five mortality in Ghana. Culture is crucial in shaping health perceptions and practices, including that of vulnerable groups such as children [45]. Thus, some ethnic groups may have cultural traditions that prioritize and promote healthy child development. Ethnic discrimination among women and children may create inferior health outcomes within the discriminated groups [46]. Also, different ethnic groups have different health-seeking behaviours such as the use of vaccinations, western/traditional medicine, birth attendance and hospitals [46]. These health-seeking behaviours and cultural practices may create different mortality rates among children of different ethnicities. Findings also indicated that children in the poorer and poorest households had a lower risk of dying before the age of five than their counterparts in the richest households, particularly in urban areas. Though this finding is counterintuitive, it is consistent with observation by [31]. The introduction and expansion of maternal and child health policies and programs in Ghana can be pivotal in improving the survival of children in poorer households [31]. Some of these policies include the Free Maternal Health care, training and monitoring of traditional birth attendants [47, 48], the expansion of the National Health Insurance Scheme, and immunization against common childhood ailments [31]. More so, poor households in urban centres are often characterized by unhygienic conditions [49], exposing children to diseases and infections. Paradoxically, repeated exposure can sometimes lead to more resilient immune systems among children living in poorer households compared to children living in wealthier households.

While the findings from this study make a valuable contribution to the literature and health policy on under-five mortality in Ghana and similar contexts in SSA, the study has some noteworthy limitations. First, the measure of timing to under-five mortality was self-reported by mothers. Thus, there is a likelihood of recall bias. Since under-five mortality was based on the mother’s recall of previous months (birth or death month of child), it is possible some mothers could not remember the exact month of child death, especially in rural areas where birth and death certifications are rare. However, the GMHS survey follows a thorough research protocol with strict adherence to ethical guidelines, including complete disclosures (e.g., no immediate financial or other benefits to participants). Second, there is a likelihood of overstatement or understatement of under-five mortality or the age of under-five mortality in anticipation of some benefit.

## Conclusion

Using survival analysis, this study examined the geography of time to under-five mortality in Ghana. The finding showed regional and residential disparities in timing to under-five mortality in Ghana. The sex of child, mother’s age, mother’s use of internet, number of household members, ethnicity, and wealth were significant in explaining some of the geographical disparities in under-five mortality in Ghana. The findings provide salient indicators for precision and contextual child healthcare delivery in Ghana. Healthcare programs and policies must look beyond the simple residential or regional disparities in child healthcare delivery. This study provides a more nuanced approach to understanding how regional vulnerabilities intersect with residential vulnerabilities to create a disproportionate burden of under-five mortality in specific geographic and administrative regions of Ghana. Healthcare policies and programs such as immunizations and provision of basic and affordable child healthcare services should be prioritized especially in rural areas of regions with a high risk of child mortality. There is a need to also improve healthcare delivery in urban areas, particularly in northern Ghana, where deplorable healthcare service infrastructure and delivery coupled with high poverty rates put children at risk of dying before their fifth birthday. Given the inaccessibility of quality healthcare services in most regions, it is imperative for a more equitable distribution of quality healthcare services to minimize both rural and urban health risks that contribute to under-five mortalities. To effectively mitigate under-five mortality in Ghana, health policies and programs must also consider contextual socio-economic, cultural, and biological variables such as sex of the child, mother’s age, use of internet, family size and number of people living with children.

## Supporting information

S1 Table Mortality table of risk and survival rates of under 5 mortality in Ghana. (DOCX)

S2 Table Mortality table of survival rates of under-five mortality in Ghana. (DOCX)
