# Supplementary material for: Geographic disparities in the time to under-five mortality in Ghana
Source: PLoS One. 2023 Sep 12;18(9):e0291328. doi: 10.1371/journal.pone.0291328 (PMC10497139; doi:10.1371/journal.pone.0291328)
Supplement: S1 Table — (DOCX) [file pone.0291328.s001.docx]

|  | **Time (Month)** | **No. Risk** | **No. Events** | **Survival** | **Std. Error** | **95% CI** |
| --- | --- | --- | --- | --- | --- | --- |
| **Overall** |  |  |  |  |  |  |
|  | 0 | 4785 | 1860 | 0.611 | 0.007 | 0.597-0.625 |
|  | 12 | 1725 | 1466 | 0.304 | 0.006 | 0.292-0.318 |
|  | 24 | 1151 | 830 | 0.131 | 0.004 | 0.122-0.141 |
|  | 36 | 629 | 338 | 0.060 | 0.003 | 0.054-0.068 |
|  | 48 | 291 | 194 | 0.020 | 0.002 | 0.016-0.024 |
|  | 59 | 97 | 97 | 0.000 | - | - |
| **Rural** |  |  |  |  |  |  |
|  | 0 | 1330 | 483 | 0.636 | 0.013 | 0.611-0.663 |
|  | 12 | 526 | 407 | 0.330 | 0.012 | 0.306-0.357 |
|  | 24 | 361 | 260 | 0.135 | 0.009 | 0.118-0.155 |
|  | 36 | 180 | 102 | 0.058 | 0.006 | 0.047-0.072 |
|  | 48 | 78 | 55 | 0.017 | 0.003 | 0.011-0.025 |
|  | 59 | 23 | 23 | 0.000 | - | - |
| **Urban** |  |  |  |  |  |  |
|  | 0 | 1408 | 580 | 0.588 | 0.013 | 0.562-0.614 |
|  | 12 | 473 | 413 | 0.294 | 0.012 | 0.271-0.319 |
|  | 24 | 317 | 240 | 0.124 | 0.008 | 0.108-0.142 |
|  | 36 | 175 | 90 | 0.060 | 0.006 | 0.049-0.074 |
|  | 48 | 85 | 54 | 0.022 | 0.003 | 0.015-0.031 |
|  | 59 | 31 | 31 | 0.000 | - | - |
