# Supplementary material for: Geographic disparities in the time to under-five mortality in Ghana
Source: PLoS One. 2023 Sep 12;18(9):e0291328. doi: 10.1371/journal.pone.0291328 (PMC10497139; doi:10.1371/journal.pone.0291328)
Supplement: S2 Table — (DOCX) [file pone.0291328.s002.docx]

| **Region** | **Time (Months)** | **No. Risk** | **No. of events** | **Survival** | **Std. Error** | **95% CI** |
| --- | --- | --- | --- | --- | --- | --- |
| **Greater Accra** |  |  |  |  |  |  |
|  | 0 | 208 | 107 | 0.485 | 0.034 | 0.422-0.580 |
|  | 12 | 49 | 56 | 0.216 | 0.028 | 0.167-0.280 |
|  | 24 | 35 | 27 | 0.088 | 0.194 | 0.055-0.134 |
|  | 36 | 18 | 7 | 0.052 | 0.015 | 0.029-0.094 |
|  | 48 | 11 | 9 | 0.009 | 0.006 | 0.002-0.038 |
|  | 59 | 2 | 2 | 0.000 | - | - |
| **Western** |  |  |  |  |  |  |
|  | 0 | 360 | 153 | 0.575 | 0.026 | 0.526-0.624 |
|  | 12 | 121 | 106 | 0.280 | 0.023 | 0.237-0.331 |
|  | 24 | 74 | 59 | 0.116 | 0.016 | 0.087-0.155 |
|  | 36 | 42 | 26 | 0.044 | 0.010 | 0.027-0.071 |
|  | 48 | 17 | 9 | 0.019 | 0.007 | 0.009-0.040 |
|  | 59 | 7 | 7 | 0.000 | - | - |
| **Eastern** |  |  |  |  |  |  |
|  | 0 | 307 | 138 | 0.550 | 0.028 | 0.497-0.609 |
|  | 12 | 89 | 86 | 0.270 | 0.025 | 0.224-0.324 |
|  | 24 | 69 | 46 | 0.120 | 0.018 | 0.089-0.163 |
|  | 36 | 37 | 21 | 0.052 | 0.012 | 0.032-0.084 |
|  | 48 | 16 | 12 | 0.013 | 0.006 | 0.004-0.034 |
|  | 59 | 4 | 4 | 0.000 | - | - |
| **Central** |  |  |  |  |  |  |
|  | 0 | 330 | 137 | 0.584 | 0.027 | 0.534-0.640 |
|  | 12 | 103 | 105 | 0.266 | 0.024 | 0.222-0.318 |
|  | 24 | 56 | 56 | 0.097 | 0.016 | 0.069-0.134 |
|  | 36 | 32 | 16 | 0.048 | 0.011 | 0.030-0.078 |
|  | 48 | 16 | 11 | 0.015 | 0.006 | 0.007-0.036 |
|  | 59 | 5 | 5 | 0.000 | - | - |
| **Volta** |  |  |  |  |  |  |
|  | 0 | 259 | 121 | 0.532 | 0.031 | 0.475-0.597 |
|  | 12 | 77 | 71 | 0.258 | 0.027 | 0.210-0.317 |
|  | 24 | 54 | 34 | 0.127 | 0.020 | 0.092- 0.175 |
|  | 36 | 33 | 14 | 0.073 | 0.016 | 0.047-0.113 |
|  | 48 | 19 | 17 | 0.007 | 0.005 | 0.001-0.030 |
|  | 59 | 2 | 2 | 0.000 | - | - |
| **Ashanti** |  |  |  |  |  |  |
|  | 0 | 443 | 206 | 0.535 | 0.023 | 0.490-0.583 |
|  | 12 | 125 | 132 | 0.237 | 0.020 | 0.200-0.280 |
|  | 24 | 81 | 66 | 0.088 | 0.013 | 0.065-0.118 |
|  | 36 | 39 | 20 | 0.042 | 0.009 | 0.027-0.066 |
|  | 48 | 19 | 11 | 0.018 | 0.006 | 0.009-0.035 |
|  | 59 | 8 | 8 | 0.000 | - | - |
| **Brong Ahafo** |  |  |  |  |  |  |
|  | 0 | 394 | 140 | 0.644 | 0.024 | 0.599-0.693 |
|  | 12 | 151 | 124 | 0.329 | 0.023 | 0.286-0.379 |
|  | 24 | 96 | 79 | 0.129 | 0.016 | 0.100-0.167 |
|  | 36 | 51 | 23 | 0.071 | 0.012 | 0.049-0.101 |
|  | 48 | 28 | 18 | 0.025 | 0.007 | 0.013-0.046 |
|  | 59 | 10 | 10 | 0.000 | - | - |
| **Northern** |  |  |  |  |  |  |
|  | 0 | 1182 | 408 | 0.654 | 0.013 | 0.628-0.682 |
|  | 12 | 491 | 356 | 0.353 | 0.013 | 0.327-0.382 |
|  | 24 | 343 | 227 | 0.161 | 0.010 | 0.141-0.184 |
|  | 36 | 191 | 114 | 0.065 | 0.007 | 0.052-0.080 |
|  | 48 | 77 | 53 | 0.020 | 0.004 | 0.013- 0.030 |
|  | 59 | 24 | 24 | 0.000 | - | - |
| **Upper West** |  |  |  |  |  |  |
|  | 0 | 776 | 259 | 0.666 | 0.016 | 0.633-0.700 |
|  | 12 | 315 | 270 | 0.318 | 0.016 | 0.287-0.352 |
|  | 24 | 201 | 145 | 0.131 | 0.012 | 0.109-0.157 |
|  | 36 | 102 | 51 | 0.065 | 0.008 | 0.050-0.085 |
|  | 48 | 51 | 31 | 0.025 | 0.005 | 0.016-0.039 |
|  | 59 | 20 | 20 | 0.000 | - | - |
| **Upper East** |  |  |  |  |  |  |
|  | 0 | 526 | 191 | 0.636 | 0.020 | 0.597-0.679 |
|  | 12 | 204 | 160 | 0.332 | 0.020 | 0.294-0.375 |
|  | 24 | 142 | 91 | 0.159 | 0.015 | 0.131-0.194 |
|  | 36 | 84 | 46 | 0.072 | 0.011 | 0.053-0.098 |
|  | 48 | 38 | 23 | 0.028 | 0.007 | 0.017-0.047 |
|  | 59 | 15 | 15 | 0.000 | - | - |
